# Use of mefloquine in children - a review of dosage, pharmacokinetics and tolerability data

**DOI:** 10.1186/1475-2875-10-292

**Published:** 2011-10-07

**Authors:** Patricia Schlagenhauf, Miriam Adamcova, Loredana Regep, Martin T Schaerer, Sudhir Bansod, Hans-Georg Rhein

**Affiliations:** 1 University of Zurich Centre for Travel Medicine, Hirschengraben 84, 8001 Zürich, Switzerland; 2 F. Hoffmann-La Roche, 4070 Basel, Switzerland

## Abstract

**Background:**

Use of anti-malarial medication in children is hampered by a paucity of dosage, pharmacokinetic and tolerability data.

**Methods:**

Data on the use of mefloquine in children, particularly in young children weighing less than 20 kg, were reviewed using PubMed literature and reports on file.

**Results:**

Chemoprophylaxis data: Two studies with a total of 170 children were found. A simulated mefloquine plasma profile showed that doses to achieve protective chemoprophylaxis blood concentration of mefloquine of approximately 620 ng/mL (or 1.67 μmol/L) in children should be at least 5 mg/kg. This simulated plasma profile in children corresponds to that seen in adult travellers using a weekly prophylaxis dose of 250 mg. This reinforces current practice of using weight-based dosage for children. Clearance per body weight is higher in older children. For children who travel to malaria risk areas tablets can be broken and crushed as required. It is necessary to disguise the bitter taste of the drug.

Treatment data: Mefloquine treatment (alone or in combination) data are available for more than 6000 children of all age and weight categories. The stereoselectivity and pharmacokinetic profile of mefloquine in children is similar to that observed in adults. There is higher clearance in older children (aged 5-12 years) compared to younger children (aged 6-24 months). Mefloquine treatment is well tolerated in infants (5-12 kg) but vomiting is a problem at high doses. This led to the use of a "split dose" regimen with 15 mg/kg initially, followed 12 hours later by 10 mg/kg.

Mefloquine 125 mg has been used as intermittent preventive treatment (IPT) and was found to be efficacious in reducing episodes of malaria in a moderate-transmission setting but vomiting was a problem in 8% of children aged 2-11 months.

Mefloquine is also used as a component of artemisinin combination therapy (ACT) in small children. The combination artesunate plus mefloquine is a WHO approved first-line treatment for uncomplicated malaria in Africa.

**Conclusion:**

Currently available data provide a scientific basis for the use of mefloquine in small children in the chemoprophylaxis setting and as a part of treatment regimens for children living in endemic areas.

## Background

### Chemoprophylaxis

More than 900 million international arrivals were registered in 2008 and it is estimated that 7-10% of all travellers are children. *Plasmodium falciparum *malaria in a young child is a life threatening disease. Early symptoms are often atypical and difficult to recognise and in non-immune children who travel to malaria endemic areas, life-threatening complications can occur within hours of the initial symptoms [1]. Parents are advised not to travel to malaria endemic areas with small children but in reality, children comprise one in 10 of all travellers. It is essential to prevent malaria with personal protection measures and the use of an effective chemoprophylaxis in high-risk areas.

Currently, the majority of European countries, the United States of America (USA), Australia, Japan and other industrialised countries are classified as malaria non-endemic but need guidelines and effective prevention for travellers. Overall, in Europe, between 15% and 20% of all imported malaria cases are in children. An analysis of more than 17,000 cases of imported paediatric malaria [2] showed that *P. falciparum *was the dominant imported species in children and that more than 75% of all paediatric cases with known place of acquisition were acquired in Africa. At highest risk are children of immigrant families visiting friends and relatives (VFR), particularly those travelling to sub-Saharan Africa, because they tend to visit high-risk destinations for prolonged periods, they lack immunity and often do not use chemoprophylaxis or preventive measures [3] and travel medicine needs to target this group of travellers [4]. The high cost of some anti-malarial medication is also a prohibitive factor for many VFR families. Malaria is thus a real threat to tourist and child expatriates visiting or living in malaria endemic areas.

All age groups of child travellers need prophylaxis including breast-fed infants, small children through to older children until young adults aged 18 years old. Mefloquine is an effective drug for malaria prevention in Africa and has the convenience of once weekly administration. There is however some uncertainty regarding the dosage of mefloquine for chemoprophylaxis in very young children and this topic is addressed here.

### Treatment

There is also a need for a concise overview on data regarding mefloquine treatment in children, particularly those aged < 5 years, who live in endemic areas and who bear the main burden of malaria. It has been estimated that 80% of malaria deaths occur in young children in Sub Saharan Africa. Mefloquine has been used recently in Africa for intermittent preventive treatment (IPTi) of malaria in infants and as a component of artemisinin combination treatment (ACT).

This review presents a summary of pharmacokinetic, dosing and tolerability data on the use of mefloquine chemoprophylaxis and treatment in children.

## Synthesis of evidence

The data presented here were compiled from searches of PubMed through January 30th, 2011 using the search terms (alone or in combination) "mefloquine" and "children", "pharmacokinetics", "dosage", "safety" or "tolerability", "adverse event", "efficacy". The authors also searched their own documentation and obtained data on file from F. Hoffmann-La Roche previously submitted to Health Authorities.

A total of 445 papers in English, French or German were screened for data, particularly for data on mefloquine use in small children (< 20 kg). Data from 41 studies were included in the tables and manuscript. The quality of the papers chosen was not assessed i.e. the selection was not restricted to randomised, double-blind studies. All studies were included that were judged to have clinically useful data on dosage, pharmacokinetics and tolerability in small children. All regimen dosages and combinations with other medication were considered. A compilation table of international guidelines on the use of mefloquine chemoprophylaxis and stand-by treatment for children visiting malaria endemic areas was also created to reflect expert opinion in the travel medicine context worldwide.

## Discussion of reviewed data

### Pharmacokinetic basis for the dosage regimen of weekly mefloquine chemoprophylaxis

The use of anti-malarial chemoprophylaxis in children is hampered by a lack of pharmacokinetic data and paediatric drug formulations. This is because small children are routinely excluded from many clinical studies due to the need for continuous sampling that is needed to determine drug concentration-time profiles. An intensive review of the literature and data on file (Table 1) revealed two studies on the use of mefloquine chemoprophylaxis in 170 children including young children weighing less than 20 kg and over 20 kg [5] (Table 1).

**Table 1:** Key studies with pharmacokinetic data on mefloquine use in children and other important clinical data on prophylaxis and therapy in children

Reference	Children Total (n)	Age (in years)	Children (n) using Mefloquine	Main findings
**CHEMOPROPHYLAXIS DATA**				

Research Report Salako, Nigeria, 1989 Data on file of F. Hoffmann-La Roche	280	6-10 y	140 (with body weight range 14-40 kg including 62 children weighing ≤ 20 kg)	Weekly 62.5 mg mefloquine or 125 mg mefloquine every two weeks (in the form of Fansimef^®^) was effective and well tolerated even in the children weighing < 20 kg. The 62.5 mg weekly dose used here is equivalent to the currently recommended quarter tablet for malaria chemoprophylaxis in this weight category

Weiss W. R. et al. [5] Daily Primaquine Is Effective for Prophylaxis against Falciparum Malaria in Kenya: Comparison with Mefloquine, Doxycycline, and Chloroquine plus Proguanil. The Journal of Infectious Diseases, 171, 1569-1575, 1995	165	9-14 y	30 (weighing 20-54 kg)	Kenyan school children aged 9-14 had lower than expected trough levels of mefloquine after standard doses (5 mg/kg/week) (mean 406 ng/mL after 6 weeks of chemoprophylaxis). This lower trough level is explained by increased mefloquine clearence in older children.

**TREATMENT DATA**				

Luxemburger C. et al. [18] Mefloquine in infants and young children. Annals of Tropical Paediatrics 16, 281-5, 1996	>500 and	<5 y	417 (with 102 children weighing 8-12 kg with mean body weight 8 kg)	No serious toxicity or adverse events. High dose of mefloquine (25 mg/kg) was associated with vomiting. Mefloquine was administered to very young children aged 3-30 months. Young age was associated with a higher risk of vomiting. Split treatment dose is recommended: 15 mg/kg initially, followed by 10 mg/kg > 12 hours later. Apart from vomiting, mefloquine was very well tolerated by young children.

Fryauff DJ, et al. [23] Mefloquine treatment for uncomplicated Falciparum malaria in young children 6-24 months of age in northern Ghana Am J Trop Med Hyg, 76(2); 224-231, 2007	186	0.5-2 y	186 (with mean body weight 8 kg)	Mefloquine single dose 20 mg/kg was evaluated in Ghanaian infants. Drug levels among infants that tolerated MQ well were not associated with age, weight or pre-existing symptoms of vomiting or diarrhea.

Bourahla A. et al. [10] Stereoselective pharmacokinetics of mefloquine in young children. European Journal of Clinical Pharmacology 50, 241-244, 1996.	12	0.5-2 y	12 (with mean body weight of 9.5 kg)	Stereoselective pharmacokinetics in children aged 6 to 24 months are similar to those observed in adults

Hellgren U. et al. [24] Standard and reduced doses of mefloquine for treatment of *Plasmodium falciparum *in Tanzania: whole blood concentrations in relation to adverse reactions, in vivo response, and in vitro tolerability. Am J Trop Med Hyg 45, 254-262, 1991	53	7-10 y	53	The dose of 6 mg/kg and higher doses eliminated *P. falciparum *parasites in children whereas a 2.5 mg/kg dose was not as effective. This supports the currently recommended 5 mg/kg dosage.

Nosten F. et al. [25] Mefloquine pharmacokinetics and resistance in children with acute falciparum malaria. Brit J Clin Pharmacol 31, 556-559, 1991	12	5-10 y	12	A single dose of 15 mg/kg led to whole blood C_max _of 2031 ug/L, t_max _mean of 8 hours (6-24) and a mean oral clearance of 0.031 L/h/kg. Comparable to adults.

Singhasivanon V. et al. [8] Pharmacokinetics of mefloquine in children aged 6 to 24 months. European Journal of Drug Metabolism and Pharmacokinetics 17, 275-279, 1992	12	0.5-2 y	12	A single dose of mefloquine 25 mg/kg led to a C_max _of 3320 ug/L, t_max _12.8 hours, elimination half-life (10.3 days), volume of distribution (12 L/kg) and AUC (35.6 mg/L/day) in children aged 6 months to 2 years. Comparable to adults.

Singhasivanon V. et al. [9] Pharmacokinetics of mefloquine in Thai children aged 5-12 years suffering from uncomplicated falciparum malaria treated with MSP or MSP plus primaquine. Eur J Drug Metab Pharmacokin 19, No 1, 27-32, 1994	18	5-12 y	18	Pharmacokinetic values in older children similar to children aged 6 months to 2 years except that clearance per body weight (0.049 L/h/kg) was higher in older children.

Some prophylaxis data on file on the use of mefloquine (in the form of the combination Fansimef^®^) is valuable in the evidence synthesis. The use of mefloquine at therapy doses has been widely evaluated in infants and supports the limited available pharmacokinetic data in the chemoprophylaxis setting (Tables 2 and 3).

**Table 2:** Recent Artemisinin Combination Treatment (ACT) Studies in small children

**Reference **[11-16]	Total No. of children	Age years/weight kg	No. of children treated with mefloquine	Important findings
Faye B et al. [11] A randomized trial of artesunate mefloquine versus artemether lumefantrine for the treatment of uncomplicated *Plasmodium falciparum *malaria in Senegalese children. Am J Trop Med Hyg 82(1), 140-144, 2010	320	4-5 y (10-20 kg)	160	The mefloquine (25 mg/kg) combination was effective > 96% and well tolerated. Even in very low weight children, vomiting in mefloquine arm was less than in comparator: 30% versus 36%

Sowunmi A et al. [12] Therapeutic efficacy and effects of artesunate-mefloquine and mefloquine alone on malaria-associated anemia in children with uncomplicated *Plasmodium falciparum *malaria in southwest Nigeria. Am J Trop Med Hyg 81(6), 979-986, 2009	342	<10 y (7-46 kg)	342	Fever and parasite clearance were faster with artesunate-mefloquine (25 mg/kg) than with mefloquine (25 mg/kg) alone. Resolution of anemia was similar in both groups. Both regimens were effective and well tolerated.

Tietche F et al. [13] Tolerability and efficacy of a pediatric granule formulation of artesunate-mefloquine in young children from Cameroon with uncomplicated falciparum malaria. Am J Trop Med Hyg 82(6), 1034-1040, 2010	213	Mean age 3 y (10-20 kg)	213	The combination was well tolerated and highly efficacious

Mayxay M et al, [14] A phase III, randomized, non-inferiority trial to assess the efficacy and safety of dihydroartemisin-piperaquine versus artesunate-mefloquine in patients with uncomplicated *Plasmodium falciparum *malaria in Southern Laos. Am J Trop Med Hyg, 83(6)1221-1229, 2010	205	< 15 y	69	Both regimens were effective, more adverse events were recorded for the AM group

Frey SG et al. [15] Artesunate-mefloquine combination therapy in acute *Plasmodium falciparum *malaria in young children: a field study regarding neurological and neuropsychiatric safety. Malaria J,9:291, 2010	220	10-20 kg	220	Mefloquine (125 mg/day) for 3 days (in combination with artesunate (50 mg/day) was well tolerated by small children with a low incidence of neurological and neuropsychiatric adverse events, mainly sleeping disorder. All events resolved spontaneously.

Ramharter M et al. [16] Pharmacokinetics of two paediatric artesunate-mefloquine drug formulations in the treatment of uncomplicated falciparum malaria in Gabon. J Antimicrob Chemother 60, 1091-1096, 2007	24	2-12 y and 11-37 kg	24	Exploratory analysis of mefloquine plasma levels showed a trend towards higher concentrations in younger age groups. All children, regardless of formulation used, achieved therapeutic and post treatment prophylactic protective levels of mefloquine

**Table 3:** Older studies prior to year 2000 documenting mefloquine use in children

Malaria treatment studies Author/Reference	Age (in years)	No. of children treated with mefloquine	Location of the study
Tin F et al. [26] Single dose treatment of falciparum malaria with mefloquine: field studies with different doses in semi-immune adults and children in Burma. Bull WHO 60, 913-917, 1982	5-12	89	Myanmar

Chongsuphajaisiddhi T. et al. [27] A phase-III clinical trial of mefloquine in children with chloroquine-resistant falciparum malaria in Thailand. Bull WHO 65, 223-226, 1987	5-12	82	Thailand

Guo X.B. [28] Double-blind dose finding study of mefloquine-sulfadoxine-pyrimethamine in children with acute falciparum malaria. Trans Roy Soc Trop Med & Hyg 82: 538-540, 1988	5-15	60	China

Sowunmi A. et al. [29] Clinical efficacy of mefloquine in children suffering from chloroquine-resistant *Plasmodium falciparum *malaria in Nigeria. Transactions of the Royal Society of Tropical Medicine & Hygiene, 84, 761-764, 1990	0.5-11	62	Nigeria

Trinh T.K. [30] Double-blind studies with mefloquine alone and in combination with sulfadoxine-pyrimethamine in 120 adults and 120 children with falciparum malaria in Vietnam. Trans Roy Soc Trop Med & Hyg 84, No 1, 50-53, 1990	6-12	80	Vietnam

Slutsker L.M. et al. [31] Mefloquine therapy for *Plasmodium falciparum *malaria in children under 5 years of age in Malawi: in vivo/in vitro efficacy and correlation of drug concentration with parasitological outcome. Bulletin of the World Health Organization 68, 53-59, 1990.	< 5	121	Malawi

Nosten F. et al. [32] Mefloquine-resistant falciparum malaria on the Thai-Burmese border. Lancet 337, 1140-1143, 1991.	< 15	245	Thai-Myanmar border

Sowunmi A. et al. [33] Evaluation of the relative efficacy of various antimalarial drugs in Nigerian children under five years of age suffering from acute uncomplicated falciparum malaria. Annals of Tropical Medicine and Parasitology 86, 1-8, 1992.	< 5	100	Nigeria

Sowunmi A. et al. [34] The relationship between the response of *Plasmodium falciparum *malaria to mefloquine in African children and its sensitivity in vitro. Trans Roy So Trop Med & Hyg 86, 368-371, 1992	4-12	85	Nigeria

Ter Kuile F. et al. [21] High-dose mefloquine in the treatment of multidrug-resistant falciparum malaria. Journal of Infectious Diseases 166, 1393-1400, 1992.	< 15	117	Thai- Myanmar border

Ter Kuile F. et al [35] Halofantrine versus mefloquine in treatment of multi-drug resistant falciparum malaria. Lancet; 341:1044-1049, 1993	< 15	95	Thai - Myanmar border

Smithuis F.M. [36] Comparison of two mefloquine regimens for treatment of *Plasmodium falciparum *malaria on the north eastern Thai-Cambodian border. Antimicrobial agents and chemotherapy, 37, No 9, 1977-1981, 1993	< 15	27	Thai-Cambodian border

Piarroux R. [37] Choice of therapy for imported cases of falciparum malaria in children: a retrospective study of 100 cases seen in Marseilles, France. Trans Roy Soc Trop Med & Hyg 87 No 1, 72-74, 1993	< 15	12	Imported paediatric malaria in France

Luxemburger C. et al. [38] Single day mefloquine-artesunate combination in the treatment of multi-drug resistant falciparum malaria. Trans Roy Soc Trop Med & Hyg, 88, 213-217, 1994.	< 15	237	Thai-Myanmar border

Sowunmi A. et al. [39] Open comparison of mefloquine, MSP and chloroquine in acute uncomplicated falciparum malaria in children. Trans Roy Soc Trop Med & Hyg, 89, 303-305, 1995.	0.5-10	43	Nigeria

Ter Kuile F. et al. [40] Predictors of mefloquine treatment failure: a prospective study of 1590 patients with uncomplicated falciparum malaria. Transactions of the Royal Society of Tropical Medicine and Hygiene 89, 660-664, 1995.	< 15	752	Thai- Myanmar border

Ter Kuile F. et al. [19] Mefloquine treatment of acute falciparum malaria: a prospective study of non-serious adverse effects in 3673 patients. Bulletin World Health Organization 73, 631-642, 1995.	< 14	1319	Thai- Myanmar border

Radloff PD. et al. [41] Arteflene compared with mefloquine for treating *Plasmodium falciparum *malaria in children. American Journal of Tropical Medicine and Hygiene, 55, 259-262, 1996	7-12	21	Gabon

Sowunmi A. et al. [42] Open comparison of artemether and mefloquine in uncomplicated *Plasmodium falciparum *hyperparasitaemia in children. Annals of Tropical Paediatrics 16, 5-9, 1996	1-10	43	Nigeria

Price RN. [43] Artesunate/mefloquine treatment of multi-drug resistant falciparum malaria. Trans Roy Soc Trop Med & Hyg 91 573-577, 1997	< 14	1453	Thai-Myanmar Border

Ranford-Cartwright LC. et al. [44] Molecular analysis of recrudescent parasites in a *Plasmodium falciparum *drug efficacy trial in Gabon. Trans Roy Soc Trop Med & Hyg 91, 719-724, 1997	< 15	64	Gabon

Okoyeh J.N. [45] Responses of multidrug-resistant *Plasmodium falciparum *parasites to mefloquine in Nigerian children. Trop Med Int Health, 2, No 4, 319-324, 1997	0.5-7	33	Nigeria

Lell B.et al. [46] Malaria chemotherapy trial at a minimal effective dose of mefloquine-sulfadoxine-pyrimethamine compared with equivalent doses of Sulfadoxine/Pyrimethamine or Mefloquine alone. Am J Trop Med Hyg 58, No 5, 619-624, 1998	< 15	76	Gabon

Luxemburger C. et al. [47] Early vomiting of mefloquine in children with malaria is not modified by the timing of antipyretic treatment. Trans Roy Soc Trop Med & Hyg, 92, 562-563, 1998	2-15	unclear	Thai- Myanmar border

**Figure 1:**
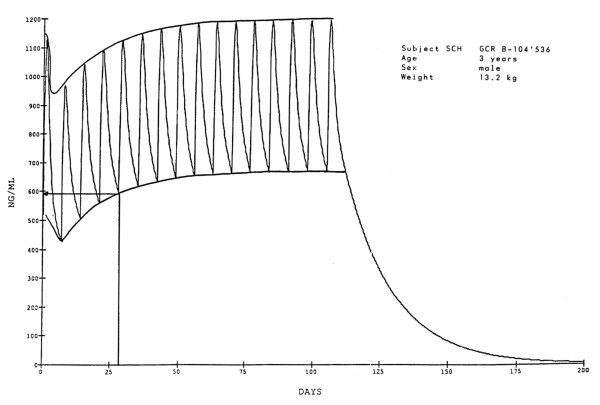
**Plasma levels during chemoprophylaxis in children**. The Figure shows the simulated plasma levels of mefloquine expected to be achieved in a child weighting 13 kg who received an initial dose of 125 mg of mefloquine and then 16 weekly prophylaxis doses of 62.5 mg mefloquine (equivalent to the currently recommended ¼ tablet for this weight category of child). Mefloquine concentrations of **620 ng/mL (= 1.67 μmol/L) **are considered to be effective against *Plasmodium falciparum *in the bloodstream.

**Figure 2:**
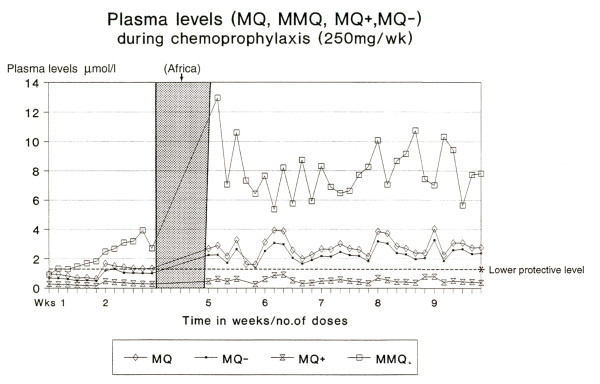
**Plasma levels achieved during chemoprophylaxis in adult travellers**. Data [52] showing plasma levels achieved during weekly prophylaxis in adult travellers, prior to travel in Africa and post travel. *Protective levels of mefloquine (620 ng/mL or 1.67 μmol/L) are achieved after two weeks of chemoprophylaxis dosing. The plasma profile in adults is similar to that in the simulated profile in children (Figure 1). MQ: mefloquine, MQ+, MQ-: mefloquine enantiomers, MMQ: carboxylic acid metabolite of mefloquine

A first study on mefloquine in a prophylactic setting (as a component of Fansimef ^®^) includes a simulated plasma profile using maintenance doses of 62.5 mg weekly mefloquine (n = 70) equivalent to ¼ of a tablet, the currently recommended chemoprophylactic dose in small children weighing 10-20 kg (Figure 1). This dosage leads to a blood concentration of mefloquine of approximately 620 ng/mL (or 1.67 μmol/L) which is considered effective against *P. falciparum *malaria [6,7]. The plasma profile in children (Figure 1), simulated for a period of 16 weeks, corresponds to that seen for adult travellers (Figure 2) using weekly prophylaxis doses of 250 mg mefloquine [6,7]. Weekly 62.5 mg mefloquine or 125 mg mefloquine every two weeks (in the form of Fansimef^®^) was effective as malaria prophylaxis and well tolerated even in the children weighing < 20 kg (Table 1).

To date, classic pharmacokinetic studies with multiple sampling and concentration time profiles during chemoprophylaxis have not been performed in small children but this is the situation with many available malaria chemoprophylaxis medications due to the need for continuous sampling.

For children who travel to malaria risk areas tablets can be broken or cut with a blade and crushed as required. It is necessary to disguise the bitter taste of the drug using chocolate or sweet yoghurt. There are no data available on the stability of mefloquine in crushed or broken tablets and cutting of the tablets is probably best done just prior to administration.

A second study in older Kenyan school children aged 9-14 [5] showed lower than expected trough levels of mefloquine after standard doses (mean 406 ng/mL after six weeks of chemoprophylaxis), which is in agreement with the Singhasivanon studies from the early 90's [8,9] that showed greater clearance of mefloquine in older children.

### Position of international expert groups with regard to mefloquine chemoprophylaxis

This pivotal position of mefloquine in the prevention of malaria in small children who travel to malaria endemic areas is reflected in the international guidelines (Table 4). The major authorities, such as WHO, CDC, DTG, UK, French and Canadian expert groups recognise mefloquine as a valuable chemoprophylaxis for small children weighing < 20 kg. For some high-risk travellers visiting friends and relatives (VFR) in their country of origin, mefloquine offers the only option for an effective, well-tolerated, chemoprophylaxis for their small children who travel to high risk malaria areas in Africa. The expert groups vary in their guidelines particularly with regard to the lower weight limit for the use of mefloquine.

**Table 4:** International guidelines showing the importance of mefloquine (MQ) for the chemoprophylaxis of malaria

Authority/Expert Group	MQ Chemoprophylaxis in children	MQ Therapy of uncomplicated malaria including Stand-by emergency self treatment (SBET) in children
WHO International Travel and Health [1]	Mefloquine recommended for chemoprophylaxis for children weighting > 5 kg. Dosage 5 mg/kg/week	Treatment of uncomplicated malaria in children weighing > 5 kg. Dosage 25 mg/kg as split dose (15 mg/kg followed by 10 mg/kg 6-24 hours apart)

DTG [48] Deutsche Tropenmed. Gesellschaft http://www.dtg.org/uploads/media/Malaria_2010.pdf	Mefloquine recommended for chemoprophylaxis for children weighing > 5 kg. Dosage 5 mg/kg/week	No longer routinely recommended for SBET

Canadian Guidelines CATMAT[49]	Mefloquine recommended for chemoprophylaxis in travellers > 5 kg body weight (5 mg/kg once weekly)-start 3 weeks before travel	Not routinely recommended for SBET
	5-10 kg 1/8 Tablet	
	10-20 kg ¼ Tablet	
	20-30 kg 1/2 Tablet	
	30-35 kg ¾ Tablet	
	> 45 kg 1 tablet	

CDC [50]	Begin 1-2 weeks before travel	Not routinely recommended for SBET
	≤ 9 kg (5 mg/kg salt once weekly)	
	> 9 kg-19 kg ¼ Tablet once weekly	
	> 19-30 kg 1/2 Tablet once weekly	
	> 31-45 kg ¾ Tablet once weekly	
	> 45 kg 1 Tablet once weekly	

UK Guidelines [51]	Weekly mefloquine	Not routinely recommended for SBET
	< 6 kg not recommended	
	6-9.9 kg ¼ Tablet weekly	
	10-15.9 kg ¼ Tablet weekly	
	16-24.9 kg 1/2 Tablet weekly	
	25-44.9 kg ¾ Tablet weekly	
	45 kg and over 1 Tablet weekly	

French Guidelines [53]	Mefloquine 5 mg/kg per week.	Not routinely recommended for SBET
	Start 10 days before travel, take throughout the exposure period and for 3 weeks thereafter	
	< 15 kg not recommended	
	15-19 kg: ¼ Tablet weekly	
	19-30 kg: 1/2 Tablet weekly	
	30-45 kg: ¾ Tablet weekly	

### Mefloquine treatment in children

Data were found on mefloquine treatment in more than 6,000 children of all ages and weights (Tables 1, 3) in Asia and Africa and data on the treatment of imported malaria in children in Marseille, France (Table 3). In these studies, mefloquine was used either alone or in combination with other anti-malarials. These findings indicate a predictable pharmacokinetic profile of mefloquine in children, which is similar to the pharmacokinetic profile of the drug observed in adults (i.e. mean absorption half-life of 2.1 hours, peak blood concentration after about 17 hours, slow systemic clearance, long elimination half-life - mean 18.1 days). The main age related difference in pharmacokinetics is that clearance per body weight is higher in older children aged 5-12 years compared to younger children aged 6 to 24 months [8,9]. A detailed pharmacokinetic study in infants with a mean weight of 9.5 kg showed that the stereoselectivity of mefloquine in children is similar to that observed in adults [10] which means that after administration of the racemic mixture, the pharmacokinetics at steady state are dominated by the (-) enantiomer as is the case with adults.

### Efficacy and tolerability of mefloquine treatment in children

Many of the early mefloquine treatment studies, prior to the year 2000 (Table 3) were conducted in children living in an area of multi-drug resistant malaria on the Thai-Myanmar border. These studies monitor the decline in the efficacy of mefloquine monotherapy in this area over time. Increasing the dose from 15 to 25 mg/kg extended the therapeutic life of the drug in the region. Currently in multiple drug resistant areas in South East Asia, monotherapy is no longer recommended by the WHO and ACT is seen as the only option. Mefloquine 25 mg/kg combined with artesunate has been extensively evaluated in recent years [11-16] (Table 2). In Africa, many countries have moved away from mono-therapy and the WHO Malaria Treatment Guidelines 2010 (2^nd ^edition) [17] recommend the combination therapy "artesunate plus mefloquine" as a first line treatment for uncomplicated malaria in Africa. The combination of a fast-acting artemisinin derivative, such as artesunate, with an anti-malarial that has sustained activity, such as mefloquine, is now an accepted approach for malaria treatment in endemic areas.

Recent studies of this ACT combination show high efficacy and good tolerability [11,12].

The early studies on mefloquine (Tables 1, 3) are however still a valuable source of information on the pharmacokinetics and tolerability of different dosages of mefloquine in children.

Luxemburger *et al *[18] reported on the use of mefloquine in more than 500 children under the age of 5 years including a total of 102 infants aged less than 30 months and weighing from five to 12 kg. This study focused on efficacy and tolerability of treatment doses of mefloquine and found that vomiting was a problem at high doses (25 mg/kg) in small children which lead to the split dosage regimen 15 mg/kg initially, followed by 10 mg/kg > 12 hours later. Otherwise, no serious toxicity was associated with mefloquine therapy (at high doses compared to low doses recommended for chemoprophylaxis) and in particular no major neuropsychiatric abnormalities were detected. The authors concluded that mefloquine (25 mg/kg) was very well tolerated in small children weighing > 5 kg except for vomiting. Vomiting of malaria treatment is important due to reduced oral bioavailability and possible subsequent treatment failure. Mefloquine related vomiting was investigated in detail between 1990 and 1995 in Thailand [19-21]. The investigators found that 30% of children aged less than 2 years vomited after high dose mefloquine treatment (25 mg/kg) but that vomiting could be reduced by 40% when the split dose regimen (15 mg/kg initially, followed by 10 mg/kg > 12 hours later) was used. It was possible to reduce mefloquine-associated vomiting by 50% by giving mefloquine on the second day in combination with artesunate. The authors concluded that high dose mefloquine was well tolerated when given as a split dose.

A more recent four-arm study of intermittent preventive treatment of malaria in infants (IPTi) in Africa compared mefloquine (125 mg), chlorproguanil (15 mg) plus dapsone (18.75 mg), sulfadoxine (250 mg) plus pyrimethamine (12.5 mg) and placebo. Mefloquine was the most efficacious drug in reducing the incidence of clinical episodes of malaria in infants but caused vomiting in 8% of children receiving this regimen [22].

With regard to tolerability, a recent paper [15] evaluated neurological and neuropsychiatric adverse events associated with artesunate-mefloquine combination treatment in young African children. Some 220 children (weighing between 10 and 20 kg) were treated with a fixed combination of artesunate (50 mg/day) and mefloquine (125 mg/day). The investigators used a standardized neurological assessment battery and found a low incidence of neurological and neuropsychiatric adverse events in small children < 20 kg who received this combination therapy. Some 50 neurological and neuropsychiatric events occurred in 28 children and 11 events in 8 patients were considered treatment related. Sleeping disorders were reported for 2.3% of children, neurological disorders in 1.4%, neuropsychiatric disorders in 1% and eating disorders in 0.5% of the children. Adverse events were of mild to moderate intensity and resolved spontaneously. In an earlier study of very young Karen children in South East Asia, neither mefloquine nor artesunate resulted in significant impairment of behavior or motor function when compared with non-febrile controls [54].

## Conclusions

There is an evidence base for the use of mefloquine both in paediatric chemoprophylaxis and in the treatment of paediatric malaria. The findings of the treatment studies done in children < 20 kg indicate a predictable pharmacokinetic profile of mefloquine in children which is similar to that observed in adults. The main age related difference in pharmacokinetics is that clearance per body weight is higher in older children aged 5-12 years compared to younger children aged 6 to 24 months. Stereoselectivity of mefloquine in children is similar to that observed in adults. Tolerability of mefloquine in small children appears superior to that in adults except for vomiting at high therapy doses, which can be mitigated by using a split dose. Two studies show a low percentage of self-limiting neurological and neuropsychiatric adverse events in children treated with artesunate/mefloquine ACT.

There are few data on chemoprophylaxis in small children but these suggest that the blood/plasma profile of mefloquine in small children is similar to that seen in adults. This dosage (for chemoprophylaxis) for children who travel to malarious areas should be at least 5 mg/kg to achieve mefloquine protective levels of 620 ng/ml in the endemic region. This reasoning is followed by expert groups in travel medicine such as the WHO with the recommendations that dosage schedules should be based on weight and that mefloquine may be given to infants of more than 5 kg body weight. There are no data on the stability of crushed or broken tablets and cutting of the tablets is probably best done just prior to administration. The bitter taste of mefloquine chemoprophylaxis should be disguised to increase adherence.

## Conflict of interest statement

This paper is based on a regulatory update done by F. Hoffmann-La Roche. PS was the external consultant who received consultancy fees for preparing the regulatory update. PS has also received research funding from Glaxo Smith Klein, F. Hoffmann-La Roche and Pfizer. PS has received speaker's honoraria from Glaxo Smith Klein, F. Hoffmann-La Roche and sigma-tau. MA, LR, MTS, SB, HGR are employees of F. Hoffmann-La Roche, Basel, Switzerland

## Authors' contributions

PS was responsible for the concept, design, acquisition of data, analysis, interpretation of the data and writing the manuscript. MA, LR, MTS, SB, HGR contributed to data acquisition, intellectual input and critically revised the paper. All authors have seen and approved the final version.
